# Editorial: Integrative Learning in US Undergraduate Public Health Education: Effective High-Impact Practices

**DOI:** 10.3389/fpubh.2019.00383

**Published:** 2019-12-11

**Authors:** Terrel L. Rhodes, Katie Darby Hein, Donna J. Petersen, Andrew Harver

**Affiliations:** ^1^Office of Quality, Curriculum and Assessment, Association of American Colleges and Universities, Washington, DC, United States; ^2^Department of Health Promotion and Behavior, University of Georgia, Athens, GA, United States; ^3^College of Public Health, University of South Florida, Tampa, FL, United States; ^4^Department of Public Health Sciences, University of North Carolina Charlotte, Charlotte, NC, United States

**Keywords:** Boyer's model of scholarship, curriculum development, high-impact practices, integrative learning, scholarship of teaching, undergraduate public health education

## Introduction

Nearly 30 years ago, Ernest L. Boyer introduced a model of scholarship specifically focused on how we know something is scholarship (worthy of our attention as educators); how curriculum is a form of scholarship (at its best reflective of the specific discipline); and what is the quality of the scholarship or practice (how do we know quality practice when we see it) (1). The former Association of Schools of Public Health subsequently embraced application of the model to understanding scholarship in public health practice (2). This current volume does not derive directly from those origins, but nonetheless represents a well-timed exploration and example of where higher education has progressed in bringing the innovative, integrative conceptualization of higher education scholarship and practice laid out by Boyer, to realization through the growing arena of undergraduate public health programs.

The articles that follow recognize the importance of faculty to achieve curricular, pedagogical, and hence student learning goals. One of the authors of Scholarship Assessed (3) stated in 1997:

“The goal…was to…give scholarship a…more efficacious meaning…a new paradigm of scholarship, one with four separate yet interlocking parts: the discovery of knowledge, the integration of knowledge, the application of knowledge, and the scholarship of teaching. The first two kinds of scholarship—the discovery and integration of knowledge—reflect the investigative and synthesizing traditions of academic life. The third element, the application of knowledge, moves toward engagement as the scholar asks, “How can knowledge be responsibly applied to consequential problems?” Finally, the scholarship of teaching recognizes that the work of the scholar becomes consequential only as it is shared with others.”

At the same time, the authors included here were invited to specifically address a second arena of scholarly practice associated with additional elements of Boyer's legacy, effective High-Impact Practices (HIPs)—practices that engage students, faculty, and often broader communities in integrative learning that connect academic and extra-academic learning environments. Research on HIPs has identified a series of criteria associated with pedagogical practices shown to be beneficial for college students from many backgrounds, especially students of color and non-traditional students who have different strengths and weaknesses compared to traditional college students (4–7). These high impact criteria include:

- Students must devote considerable time and effort to purposeful tasks that deepen student investment in the activity and connection to their academic program and college;
- Students find themselves in situations where they must interact with faculty, peers, and often community members about substantive matters over extended periods;
- Participating in one or more of these activities increases the likelihood students will experience diversity through contact with people, who are different from themselves;
- Students receive frequent feedback about their performance;
- Students see how what they are learning works in different settings on and off campus; and
- Participating in one or more of these practices in the context of a coherent, academically challenging curriculum deepens learning, brings one's values and beliefs into awareness, and develops the capacity to take the measure of events and actions and put them in perspective.

## Contributions to the Collection

The purpose of this Research Topic was to examine the role of effective HIPs within the curriculum of undergraduate public health programs—through original research, reports, and reviews—that promote integrative learning experiences. The final collection of curated contributions is distributed between two themes: (1) current application of high-impact educational practices in undergraduate public health education; and (2) the design and implementation of integrative undergraduate public curriculum and programs.

### High-Impact Educational Practices

Authentic and intentional assignments are fundamental to HIPs and encourage “integrative learning,” both a simple and complex approach to pedagogy. HIPs invariably target written and oral communication skills, teamwork, ethical decision-making, critical thinking, and the application of knowledge—skills uniformly valued in recent employer surveys [e.g., (8)]. Contributions that describe the development and assessment of high-impact educational practices on the authors' campuses include:

collaborative learning (interprofessional education);
diversity and global learning (study abroad);
ePortfolios (capstone coursework);
experiential leaning (research, internship, service learning, or global learning);
experiential learning (community-based learning);
experiential learning (service learning-introductory coursework);
experiential learning (service learning-capstone coursework); and
learning communities (cohort model).

### Integrated Public Health Curriculum and Program Development

A second set of contributions that address aspects of the design and implementation of authentic and intentional teaching and learning practices for students who enroll in one of the nation's fastest-growing majors include:

active learning and deliberative pedagogy;
integrative curriculum reform; and
student perceptions of integrative practices.

## Conclusion

High-Impact Practices are not high impact simply because they exhibit the criteria listed above. HIPs are highly effective when they are intentionally embedded into a curriculum—both formal and informal curricula—that brings students from an introduction to a field of study through iterative practice of knowledge and skills to demonstrated levels of quality warranting a credential.

Undergraduate public health programs are perfectly positioned to provide a framework for integrated learning that encompasses not only the essential learning outcomes that employers continue to demand—critical thinking, working with diverse others, written and oral communications, ethics, analysis, etc.—but a curriculum that is scaffolded and replete with opportunities to practice and enhance performance and application of knowledge and abilities to important personal, social, and global challenges and needs.

This Research Topic is situated, in part, within a confluence of initiatives and priorities that address demands for campuses to provide meaningful evidence of student learning that guides institutional decision-making to improve student performance. Monitoring and responding to student performance levels are not new activities for professional degree programs such as public health. As undergraduate public health degree programs continue to flourish at an unprecedented pace, however—for example, “the number of undergraduate degrees awarded will probably soon meet, and likely exceed, the number of graduate degrees awarded” (9)—the need for authentic and integrative student-centered practices at the undergraduate level will similarly escalate.

We concur, and close, with Scobey's (10) recent argument,

“…we want an educational future that draws on, and draws out, the implications of the new HIPs. Such a model would provide students with an arc of learning experiences—active, collaborative, boundary-crossing, and integrative—that interweave intellectual professional, civic, and personal growth.”

## Author Contributions

TR, along with KH, took the lead preparing the first draft. AH edited the first draft and added additional content to the manuscript. DP critically reviewed subsequent versions of the Editorial. All authors contributed to the Editorial and approved the final version for publication.

### Conflict of Interest

The authors declare that the research was conducted in the absence of any commercial or financial relationships that could be construed as a potential conflict of interest.
